# The Complete Maternally and Paternally Inherited Mitochondrial Genomes of the Endangered Freshwater Mussel *Solenaia carinatus* (Bivalvia: Unionidae) and Implications for Unionidae Taxonomy

**DOI:** 10.1371/journal.pone.0084352

**Published:** 2013-12-17

**Authors:** Xiao-Chen Huang, Jun Rong, Yong Liu, Ming-Hua Zhang, Yuan Wan, Shan Ouyang, Chun-Hua Zhou, Xiao-Ping Wu

**Affiliations:** 1 Center for Watershed Ecology, Institute of Life Science, Nanchang University, Nanchang, P. R. China; 2 School of Life Sciences and Food Engineering, Nanchang University, Nanchang, P. R. China; The Centre for Research and Technology, Hellas, Greece

## Abstract

Doubly uniparental inheritance (DUI) is an exception to the typical maternal inheritance of mitochondrial (mt) DNA in Metazoa, and found only in some bivalves. In species with DUI, there are two highly divergent gender-associated mt genomes: maternal (F) and paternal (M), which transmit independently and show different tissue localization. *Solenaia carinatus* is an endangered freshwater mussel species exclusive to Poyang Lake basin, China. Anthropogenic events in the watershed greatly threaten the survival of this species. Nevertheless, the taxonomy of *S. carinatus* based on shell morphology is confusing, and the subfamilial placement of the genus *Solenaia* remains unclear. In order to clarify the taxonomic status and discuss the phylogenetic implications of family Unionidae, the entire F and M mt genomes of *S. carinatus* were sequenced and compared with the mt genomes of diverse freshwater mussel species. The complete F and M mt genomes of *S. carinatus* are 16716 bp and 17102 bp in size, respectively. The F and M mt genomes of *S. carinatus* diverge by about 40% in nucleotide sequence and 48% in amino acid sequence. Compared to F counterparts, the M genome shows a more compact structure. Different gene arrangements are found in these two gender-associated mt genomes. Among these, the F genome *cox2-rrnS* gene order is considered to be a genome-level synapomorphy for female lineage of the subfamily Gonideinae. From maternal and paternal mtDNA perspectives, the phylogenetic analyses of Unionoida indicate that *S. carinatus* belongs to Gonideinae. The F and M clades in freshwater mussels are reciprocal monophyly. The phylogenetic trees advocate the classification of sampled Unionidae species into four subfamilies: Gonideinae, Ambleminae, Anodontinae, and Unioninae, which is supported by the morphological characteristics of glochidia.

## Introduction

Unionoid bivalves, known as freshwater mussels (Bivalvia: Unionoida), are one of the most important faunas in freshwater ecosystems, for their potentials to enhance biodiversity and ecosystem functioning (e.g., 1,2). The family Unionidae is the most species-rich of the Unionoida and broadly distributed across North America and Eurasia as well as tropical Mesoamerica, Africa, and southeastern Asia [3]. The Unionidae is composed of six subfamilies: Parreysiinae (Afrotropic, Indomalaya), Modellnaiinae (Indomalaya), Rectidentinae (Indomalaya), Gonideinae (Indomalaya, Palearctic, western Nearctic), Ambleminae (Nearctic, Neotropic), and Unioninae (Palearctic, Nearctic, Indomalaya), in which the latter three subfamilies account for some 551 species (82%) [3-5]. Freshwater mussels have a distinct parasitic stage in the life cycle that involves a host-fish and a highly modified larva, the glochidium [6]. They are sensitive to environmental changes, especially in the post-parasitic phase [7-9].

The global decline of freshwater mussels makes them one of the most imperiled groups of animals [10]. In China, the middle and lower reaches of the Yangtze River have the most diverse freshwater mussel fauna [11]. However, more than 80% of the unionid species in these regions are considered to be threatened or near threatened, and the dominant taxa of bivalves have shifted from large-sized unionids to the small-sized clams [12].

For conservation purposes, we first need to accurately identify unionoid bivalves and estimate genetic diversity in wild populations. Identification and classification of unionoid bivalves are usually based on adult shell morphology. However, different environmental conditions may lead to a certain degree of phenotypic plasticity in conchology. In addition, morphological characteristics of the glochidium, especially the hook type (i.e., hooked, hookless, or axe-head larvae), are very useful to phylogenetic analyses, but only for higher taxonomic categories (e.g., 13,14). On the other hand, molecular data may provide more accurate estimates of the actual phylogenetic relationships and genetic diversity in unionoid bivalves. Owing to a relatively rapid evolutionary dynamics in bivalves, analyses of mitochondrial DNA (mtDNA) sequence data could lead to satisfactory resolution [15-17].

Freshwater mussels are the only freshwater animals that transmit their mtDNA both maternally and paternally; the two other bivalves are marine mussels (Mytiloida) and marine clams (Veneroida) [18,19]. These bivalves possess a peculiar system of mtDNA transmission, termed "doubly uniparental inheritance" (DUI; [20,21]; see 22,23 for reviews) and show two highly divergent gender-associated mitochondrial genomes [16] which still obey the rule of uniparental transmission. In lineages with DUI, both sons and daughters get the F mitogenome (female-transmitted mtDNA, F genome, F-type) from their mother bivalves, as standard maternal inheritance, whereas sons only inherit and transmit the M mitogenome (male-transmitted mtDNA, M genome, M-type) of the father through sperm (e.g., [22]; but see 24-26). The maternal and paternal mitogenomes have a peculiar pattern of tissue distribution: F genome predominates in somatic tissues of both sexes and female gonadal tissues, while M genome is predominant in male gonadal tissues [23,27,28]. In the light of the DUI system, migration rates via different sexes can easily be measured and compared. The M genome can be used as a new marker for the genetic diversity estimate to be compared with the results from the F genome. The combination of F and M genomes can offer new insights into the mitogenome evolutionary history and phylogeographic structure of the freshwater mussels. Additionally, the DUI system provides an excellent model for studying basic biological questions concerning with mtDNA inheritance and the evolution of mitochondrial genomes in general [17,18,23].

Poyang Lake Basin is one of the hotspots inhabiting biodiversity of freshwater mussels in the middle and lower reaches of the Yangtze River, with approximately 75% of the unionid species endemic to China [29-32], including the exclusive species *Solenaia carinatus* (Heude). In 1877, by virtue of a fossil fragment of *S. carinatus* collected in the middle reaches of the Yangtze River, Heude [33] thought they belonged to an additional new species, and considered it a fossil species. However, over the subsequent century, the validity of this new species had not been recognized by almost all mollusk taxonomists (e.g., Haas [34]) until Liu and Wu [35] collected live specimens in Wucheng Town, Poyang Lake region, and gave a further description in 1991. Fragmentary fossil specimens of *S. carinatus* were also collected from the Quaternary of conglomerate layer in the Yellow River bank [36], indicating it once lived in northern China. Currently, due to climate and environmental changes, it only persists at extremely low numbers in Poyang Lake basin [31,32]. Like other *Solenaia* species, *S.* carinatus is directly targeted by local people as food [35]. Meanwhile, other anthropogenic events in watersheds such as sand excavation, dam construction, pollution, agriculture runoff, and overfishing of its potential host-fish greatly threaten the survival of this species. With elongate shell lacking hinge teeth, the morphologically inferred taxonomy of genus *Solenaia* is confusing (e.g., 30,33) and challenged by phylogenetic studies based on partial 16S rRNA sequences of *S. oleivora* [37] and Chinese Unionidae *cox1* sequences [38]. Consequently, prior to developing conservation recovery plans, the taxonomic status of *S. carinatus* needs to be resolved with more comprehensive analysis.

In this study, we described the first complete maternally and paternally inherited mitochondrial genomes of *S. carinatus*, and inferred the phylogenetic trees together with the published mitogenome data of unionoids, expecting the classification of unionids could reflect natural relationships with robust molecular evidence. The mitogenomes offer a number of genome-level features (e.g., gene orders, conserved gene clusters) that could be useful for phylogenetic purposes [17] and provide valuable tools for future ecological, population genetic assessments and conservation management of *S. carinatus* and the related unionoid species.

## Materials and Methods

### Ethics Statement

All necessary permits were obtained for the described field studies from the Poyang Lake Fishery Administration of Jiangxi Province, China. The handling of mussels was conducted in accordance with the guidelines on the care and use of animals for scientific purposes set by the Institutional Animal Care and Use Committee (IACUC) of Nanchang University, Jiangxi, China.

### Sample collection and DNA extraction

Living specimens (N = 4) of *Solenaia carinatus* were obtained from Poyang Lake (Jiangxi province, China) in 2011-2013 and were sexed by microscopic examination of gonadal tissues. Dissected tissues (gonadal tissue for one male and adductor muscle for one female) were chosen for subsequent DNA extractions and preserved at −80 °C. 

Total genomic DNA isolations were performed on the dissected tissues using Wizard^®^ SV Genomic DNA Purification System (Promega) according to the manufacturer's instructions.

The DNA concentration and quality of the extract were measured on the Nanodrop 2000 spectrophotometer (Thermo Scientific) and examined on an agarose gel as well.

### PCR amplification and mt genome sequencing

Universal primers were initially used to amplify and sequence short (ca. 500-1000 bp) regions of mtDNA. All obtained sequences of short fragments were BLAST against other unionoids in the GenBank to confirm the corresponding gender-associated mtDNA. Afterwards, specific primers were designed using Primer Premier 5.0 software (Premier Biosoft International).

To prepare the amplicons for primer-walking sequencing, amplification strategies were adjusted in these two mitogenomes due to the sequencing restriction of optimal amplicon length and complex structures (e.g., AT-rich, poly N, and repeated sequences).

For both female and male mtDNA, partial *cox1* and *rrnL* gene sequences were obtained initially using the universal primers of LCO1490/HCO2198 [39] and 16SarL/16SbrH [40] respectively.

For F-type mtDNA, *nad1* gene sequences were also obtained using the universal primers Leu-uurF/LoGlyR [41]. Three perfectly matching primers were then designed from these three gene sequences to amplify the F-type mitogenome in three long fragments (*cox1-rrnL*, *rrnL-nad1* and *nad1-cox1*).

**Figure 1:**
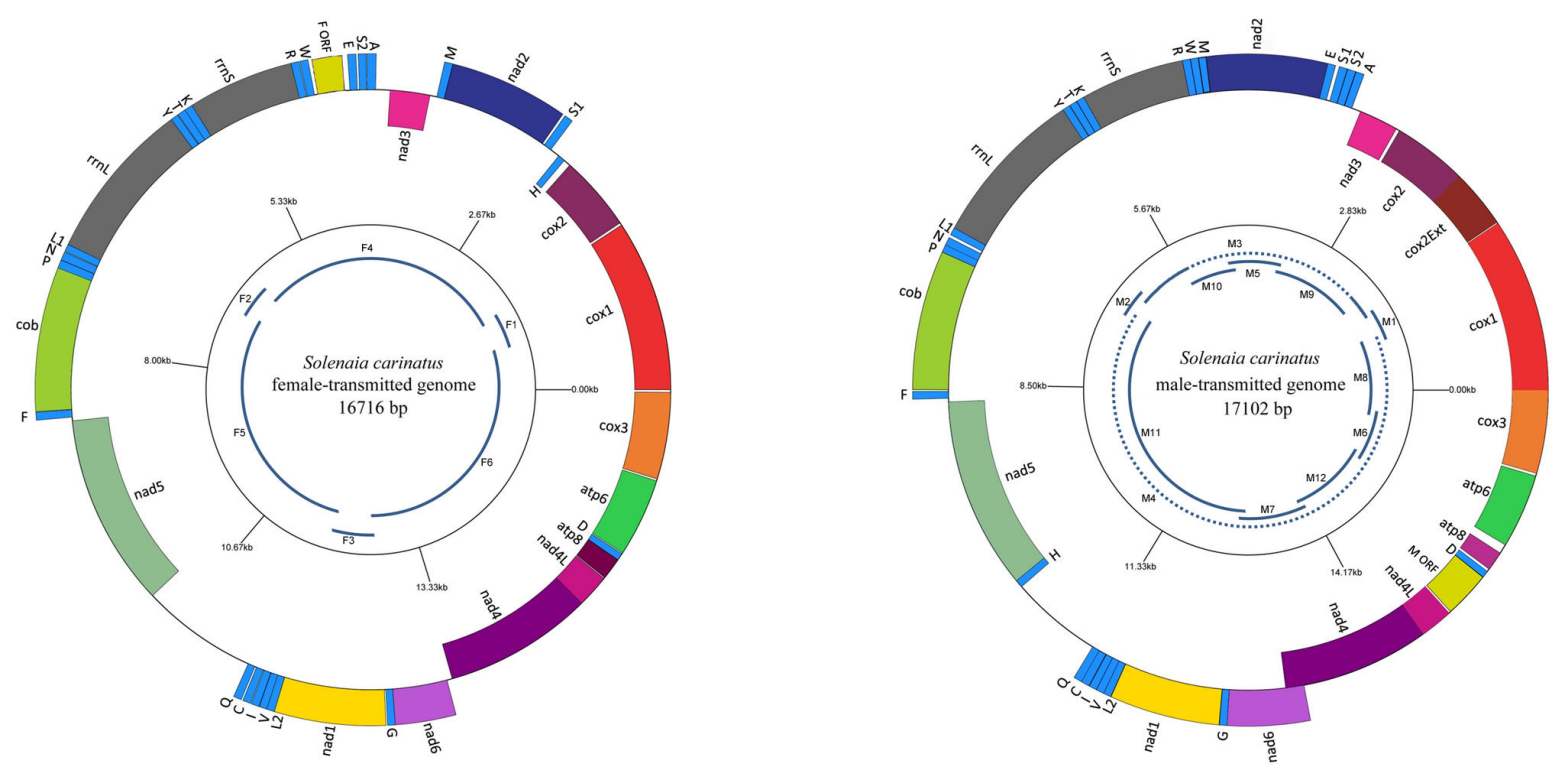
Gene maps of the F and M mitochondrial genomes of *Solenaia carinatus*. Genes illustrated on the outside of the main circle are encoded on the light (L) strand; genes on the inside of the circle are encoded on the heavy (H) strand. tRNA genes are abbreviated by one-letter code of the amino acid (S1 = tRNA^Ser^ (ucu), S2 = tRNA^Ser^ (uga), L1 = tRNA^Leu^ (uag), and L2 = tRNA^Leu^ (uaa)). The inner circle: solid lines represent the sizes and relative positions of amplified and sequenced fragments; the dotted lines represent the fragments that can be amplified but cannot be sequenced directly.

For M-type mtDNA, it was unable to amplify the *nad1* gene sequence with universal primers, which could be explained by the rapid substitution rate of the M mtDNA [42,43]. Consequently, two long-range PCR products, approximately 11 kb long *rrnL*-*cox1* and 5 kb long *cox1*-*rrnL*, were amplified but amplicons could not be sequenced or thoroughly sequenced. Subsequently, these two long fragments were used as the Sub-PCR templates to avoid coamplification of nuclear genes or contaminated F-type mitochondrial genes. Three pairs of Sub-PCR primers (to amplify the M5, M6 and M7 fragments, Table 1, Figure 1) were designed based on alignment and comparison of M-type mitogenome sequences of the *Pyganodon grandis*, *Quadrula quadrula*, and *Venustaconcha ellipsiformis* (Table 2). Five pairs of specific primers were designed according to Sub-PCR amplicon sequences and previously obtained sequences to amplify the remaining fragments from the M-type mt genome using long-range PCR or general PCR. 

**Table 1:** Primers used for PCR amplification of female and male *Solenaia carinatus* mitochondrial genomes.

**Fragment no.**	**Primer name**	**Primer sequence (5' to 3')**	**Length (bp)**
F1/M1	LCO1490	GGTCAACAAATCATAAAGATATTGG	~680
	HCO2198	TAAACTTCAGGGTGACCAAAAAATCA	
F2/M2	16SarL	CGCCTGTTTATCAAAAACAT	~500
	16SbrH	CCGGTCTGAACTCAGATCACGT	
F3	Leu-uurF	TGGCAGAAAAGTGCATCAGATTAAAGC	~1000
	LoGlyR	CCTGCTTGGAAGGCAAGTGTACT	
F4	FCOI16s-F	AAAGAGCTGGCACAAGCAAT	~5700
	FCOI16s-R	CGGTTGCACTAATGTGGATG	
F5	F16sND1-F	CACGCCAAGGAGCACAAA	~5200
	F16sND1-R	AGGGAGGAAATACATAAGAACAGGAG	
F6	FND1COI-F	CCCACAACCACTGGCTGAC	~5600
	FND1COI-R	CATCTTGCGGGTGCTTCTTC	
M3	MCOI16s-F	GCGTCACTCCGCATCCCTACAA	~6000
	MCOI16s-R	TGCCGTTCACTGGCTCCTAAT	
M4	M16sCOI-F	AGCGTGAGCGTGCTAAGGTA	~ 11000
	M16sCOI-R	ATTGTAGGGATGCGGAGTGA	
M5	MND2-F	CGTTCAACTCGTCCATCTTC	~1100
	MND2-R	TTTTGGGTTATGAGCCCACT	
M6	MA8C3-F	AACAGGACTAAGTTGAGGCA	~1100
	MA8C3-R	GGTTTGACCTCCTGTTGG	
M7	MN6N4-F	ACCATGTACAATTGCCTTCC	~1500
	MN6N4-R	GGTATCAGCCAGAGCGATT	
M8	MC3C1-F	CAAATAAGGCACTCACAGC	~1600
	MC3C1-R	TTGTTAGGTTGGTCGATGT	
M9	MC2N2-F	AAATGCCTCTACTGGAGAAT	~1600
	MC2N2-R	GGCTTGAAAGTCCGATGT	
M10	MN212S-F	GATTCCTCACCTGGCTCAC	~1000
	MN212S-R	GGCTGCTGGCACCATTTT	
M11	M16SN64-F	GGGGCAACCTTGGAGCA	~5800
	M16SN64-R	GGGCGACAGCGATGAGAT	
M12	MN64A-F	ACCTTCCACCCAGACCTCA	~2100
	MN64A-R	CCCAGTTACTTTGGCTGTTCG	

**Table 2:** Mitochondrial genomes used in analyses.

**Order**	**Family**	**Species**	**Gender**	**Accession number**	**Reference**
Unionoida	Unionidae	*Solenaia carinatus*	F	KC848654	This study
			M	KC848655	This study
		*Lampsilis ornata*	F	NC_005335	Serb & Lydeard, 2003
		*Unio pictorum*	F	NC_015310	Soroka et al., 2010
		*Cristaria plicata*	F	NC_012716	Jiang et al., 2010
		*Anodonta woodiana*	F	HQ283346	Soroka, 2010
		*Hyriopsis schlegelii*	F	NC_015110	Lin et al. Unpublished
		*Hyriopsis cumingii*	F	NC_011763	Zheng et al. Unpublished
		*Unio japanensis*	F	AB055625	Okazaki. Unpublished
			M	AB055624	Okazaki. Unpublished
		*Pyganodon grandis*	F	NC_013661	Breton et al., 2009
			M	FJ809755	Breton et al., 2009
		*Venustaconcha ellipsiformis*	F	FJ809753	Breton et al., 2009
			M	NC_013659	Breton et al., 2009
		*Quadrula quadrula*	F	NC_013658	Breton et al., 2009
			M	FJ809751	Breton et al., 2009
		*Utterbackia peninsularis*	F	HM856636	Breton et al., 2011
			M	NC_015477	Breton et al., 2011
		*Utterbackia imbecillis*	H	NC_015479	Breton et al., 2011
		*Toxolasma parvus*	H	NC_015483	Breton et al., 2011
		*Lasmigona compressa*	H	NC_015481	Breton et al., 2011
	Margaritiferidae	*Margaritifera falcata*	H	NC_015476	Breton et al., 2011
Mytiloida	Mytilidae	*Mytilus edulis*	F	NC_006161	Boore et al., 2004
			M	AY823624	Breton et al., 2006

F-female, M-male, H-hermaphrodite (hermaphroditic F-like haplotype)

All the PCR and sequencing strategies were outlined in Figure 1. A full list of primers is presented in Table 1.

TaKaRa Ex Taq polymerase was used to amplify short fragments (<2 kb), and TaKaRa LA Taq polymerase was used to amplify long-range PCR products (>2 kb). PCR conditions were optimized for each reaction, with the annealing temperature adjusted to suit the used primers, extension time set to 1 min per kb of expected product size, and proper cycles adjusted depending upon the amplification efficiency of the primers.

General PCR conditions for Ex Taq were 98 °C for 10 s, followed by 35 cycles of 94 °C for 1 min, 50 °C for 1 min, 72 °C for 1-2 min, and a final extension of 72 °C for 7 min.

The long-range PCR was performed with the following amplification profile: 94 °C for 2 min, followed by 25-35 cycles of 94 °C for 30 s, 55 °C for 30 s, 68 °C for 5-11 min, and a final extension of 72 °C for 10 min.

Mitogenome data were generated using the primer-walking method. PCR products were purified with the SanPrep spin column DNA gel extraction kit or SanPrep spin column PCR product purification kit (Sangon Biotech), and directly sequenced using BigDye Terminator Sequencing Kit and ABI 3730xl DNA Analyzer (Applied Biosystems). 

### Gene annotation and mitogenome analysis

Sequences were assembled using SeqMan program (DNAstar). Protein-coding genes (PCGs) were initially identified using ORF Finder (http://www.ncbi.nlm.nih.gov/projects/gorf) using the invertebrate mitochondrial code and confirmed by blastp and tblastn searches. Transfer RNA genes were inferred with ARWEN [44] and MITOS [45]. Ribosomal RNA genes were identified by homology to mitochondrial rRNA genes of other unionoids, and the ends of *rrnL* and *rrnS* genes were assumed to extend to the boundaries of their flanking genes. 

The tandem repeats of the whole mitogenome were detected using mreps [46]. Secondary structures of CR regions were predicted in RNAfold WebServer [47] using minimum free energy (MFE) and partition function. Mitochondrial genomes were drawn using GenomeVx [48] followed by manual modification.

Nucleotide composition and codon usage were analyzed with MEGA 5.0 [49]. Strand asymmetry was measured using the formulas GC skew = (G - C)/(G + C) and AT skew = (A - T)/(A + T) [50]. To analyze nucleotide and amino acid sequence variability, pairwise p-Distances (pD) were used and computed with MEGA 5.0 [49]. The divergence of protein genes in synonymous (Ks) and non-synonymous (Ka) sites was calculated with PAML 4.7 [51] using Nei-Gojobori method [52].

### Sequence alignment and phylogenetic analyses

In order to elucidate the phylogenetic positions of *S. carinatus* within Unionoida, all 20 currently available complete F and M mitochondrial genomes of 15 unionoid species were downloaded from the GenBank database (Last accession to databases was in May 2013) (Table 2) and combined with sequences from this study for phylogenetic analyses, using female and male marine mussel *Mytilus edulis* as outgroup taxa. Sequences of 12 PCGs except gender-specific ORFs and *atp8* (due to its absence or constant annotation error in many bivalves [16,53]) were used in phylogenetic analyses. Each 12 PCGs was translated into amino acid sequence using the invertebrate mitochondrial genetic code in MEGA 5 [49], and aligned based on the amino acid sequence using the built-in MUSCLE [54] program with default settings, then the corresponding nucleotide sequences were retro-aligned. Alignments of sequences were manually checked and areas of ambiguous alignment as well as stop codons were excluded. The resulting alignments of trimmed nucleotides or amino acids were concatenated in SequenceMatrix [55] into two datasets with 10200 nucleotides or 3400 amino acids. To assess if the third codon position has saturation that's masking the signals required for a better resolution of the deep nodes, Mesquite 2.75 [56] was used to exclude the third codon positions and produced a new dataset with 6800 nucleotides. To sum up, three datasets were used for phylogenetic analyses: 1) amino acid sequences of 12 PCGs (AA); 2) nucleotide sequences of 12 PCGs (PCG123); 3) the first and the second codon positions of 12 PCGs (PCG12).

MrModeltest [57] and ProtTest [58] were used to select optimal substitution models for nucleotide sequence datasets and amino acid sequence alignment respectively, according to the Akaike Information Criterion (AIC). 

The Bayesian phylogenetic analyses were performed using MrBayes 3.2.1 [59]. PCG123 and PCG12 were partitioned by codon sites using GTR+I+G model, while AA using JTT model. Two sets of four chains were conducted to run simultaneously for 1 million generations and trees were sampled every 1000 generations, with a burnin of 25%. Stationarity was considered to be reached when the average standard deviation of split frequencies was less than 0.01.

The codon site based partitioned ML analyses were performed for PCG123/PCG12 in RAxML [60] implemented in raxmlGUI v.1.3 [61], using GTRGAMMAI model of nucleotide substitution with the search strategy set to rapid bootstrapping. ML analysis for AA was performed using PhyML 3.0 online execution [62], with the best chosen amino acid model JTT+I+G+F. Bootstrap supports for ML trees were calculated using 1000 bootstrap replicates for all datasets.

## Results and Discussion

### General features of the mitochondrial genomes

The maternal and paternal mt genomes of *S. carinatus* are 16716 and 17102 bp in length, respectively (see Table 3). Sequences are available in GenBank (Accession Number: KC848654 and KC848655). Differences of the genome sizes are mainly due to unassigned regions and *cox2*. Both of the newly sequenced genomes consist of 13 protein-coding genes for subunits of the respiratory chain complexes, 1 novel gender-specific ORF (i.e., female-specific FORF and male-specific MORF), 2 rRNA genes and 22 tRNA genes. In both F and M mitogenomes, 11 out of typical 37 genes are encoded on the heavy strand, and the remaining 26 genes are on the light strand (Figure 1).

**Table 3:** Main structural features of the female and male *Solenaia carinatus* mitochondrial genomes.

	*S. carinatus* F mt genome	*S. carinatus* M mt genome
Total size	16716	17102
No. of gene overlapping	2	6
Size range of gene overlapping	1 to 8	1 to 168
CR	1049	848
*rrnS*	857	857
*rrnL*	1296	1313
*cox1*	1545	1602
	(TTG/TAG)	(TTG/TAG)
*cox2*	681	1224
	(ATG/TAA)	(ATG/TAA)
*cox3*	780	774
	(ATG/TAA)	(ATT/TAG)
*nad1*	897	909
	(ATC/TAA)	(ATA/TAA)
*nad2*	963	996
	(ATG/TAA)	(TTG/TAA)
*nad3*	357	360
	(ATG/TAG)	(ATG/TAG)
*nad4*	1350	1374
	(ATT/TAG)	(TTG/TAG)
*nad4L*	297	300
	(ATG/TAG)	(ATT/TAG)
*nad5*	1734	1764
	(ATG/TAA)	(GTG/TAG)
*nad6*	489	681
	(ATC/TAA)	(ATC/TAG)
*cob*	1161	1149
	(ATC/TAG)	(ATG/TAG)
*atp6*	708	684
	(ATG/TAG)	(ATG/TAG)
*atp8*	198	177
	(GTG/TAG)	(ATG/TAG)
Gender-specific ORF	261	435
	(ATA/TAA)	(ATA/TAA)

For each protein coding genes, start and stop codons are presented in parentheses. Gene lengths are in bp.

Overlapping adjacent genes are common in unionoid mt genomes. Comparing these two newly sequenced sex-linked mt genomes, the M genome shows a more compact structure than the F genome, having more overlapping genes (6 versus 2 overlapping genes), evidently longer PCGs (*cox2* and *nad6*) and smaller CR (Figure 1, Table 3).

*nad4L* and *nad4* overlap in both F and M mt genomes, which is the case for most sequenced unionoid mt genomes [16,43,63-66]. In addition, in *S. carinatus* M genome, protein-coding gene *nad6* overlaps with *nad4* and *cox3* with *cox1*, sharing 168 and 8 bp sequences, respectively. The unusual overlap of *nad6* and *nad4* is also found in M genome of *Unio japanesis*, but only 4 bp were shared. However, the overlap of *cox3* and *cox1* has not been found in other unionoid mitogenomes so far. In F mitogenome, only one tRNA gene *trnP* overlaps with a protein-encoding gene *cob*. While in M mitogenome, not only tRNA genes overlap with PCG (*trnM-nad2*) but also different tRNA genes overlap with each other (*trnN-trnP, trnQ-trnC*).

### Protein-coding genes

Gender-specific novel mitochondrial ORFs were already described and confirmed in DUI mtDNAs (Mytilidae: [67], Unionidae: [18], and Veneridae: [68]). In unionids, gender-specific ORFs are localized in the second largest unassigned region of F and M mtDNA [18]. In all the analyzed DUI *Mytilus* species, however, the novel FORF is found in the largest unassigned region (i.e., the control region) [67].

In *S. carinatus* mitogenomes, FORF is found in F *trnE-trnW* intergenic region on the light strand, while MORF is located in M *nad4L-trnD* unassigned region on the heavy strand. FORF sequence is 261 bp long (86 aa), while MORF is 435 bp (144 aa) (Table 3). Testcode [69] predictions of coding function for the S. *carinatus* FORF and MORF sequences are all very high. The testcode scores are 0.997 for FORF and 1.224 for MORF, which indicate that these ORFs are coding with the probability of 92% and 100% respectively. These sequences do not show homology with any known proteins using BLAST Tools; therefore, the precise function of these ORFs remains unclear.

In *Venustaconcha ellipsiformis*, the existence of FORF and MORF was shown by Western blot analysis [18], and the FORF protein was not only present in mitochondria but also in the nuclear membrane and in the nucleoplasm of eggs [43]. These gender-specific ORFs may have a role in the maintenance of sperm mitochondria during embryo development, possibly masking them from the degradation processes [70].

Mitochondrial genomes often contain a variety of nonstandard initiation codons. In the invertebrate mt genomes, there are three conventional start codons (ATG, ATA, and ATT) and three alternative start codons (ATC, TTG, and GTG) [71].

In the S. *carinatus* M genome, 9 out of 14 PCGs use conventional start codon (ATG, N = 5; ATA, N = 2; ATT, N = 2) and 5 with three alternative start codons (ATC, N = 1; TTG, N = 3; GTG, N = 1) (Table 3). In F genome, except for FORF (ATA), *nad4* (ATT), *cob* (ATC), *nad1* (ATC), *nad6* (ATC), *cox1* (TTG) and *atp8* (GTG), 7 out of 14 PCGs start with orthodox ATG (Table 3).

Both F and M genome PCGs have complete termination codon TAG or TAA, with TAG occurs ten times in M and seven times in F mtDNA (Table 3). Being compared to the available bivalve mitogenomes from GenBank, *S. carinatus* M genome has the longest *cox1* (1602 bp), *nad2* (996 bp) in unionoid bivalves and the longest *nad6* (681 bp) in bivalves, while F genome has the longest *cob* (1161 bp) in freshwater mussels.

### Transfer RNA and ribosomal RNA genes

Both of the obtained *S. carinatus* F and M mt genomes contain 22 tRNA genes, including two serine tRNA and two leucine tRNA. Most of the tRNA genes are located on the light strand, except for the *trnH* and *trnD*. They vary from 61 (*trnL1*, *trnG*) to 74 (*trnA*) bp in F genome and 62 (*trnY*) to 71 (*trnH*) bp in M genome.

In both sexes, *rrnS* and *rrnL* are encoded on the light strand and are separated by *trnK*, *trnT* and *trnY* (Figure 1), as in all the unionoid mt genomes studied so far. For female and male mt genome of *S. carinatus*, respectively, the lengths of *rrnS*/*rrnL* are 857/1296 and 857/1313 bp (Table 3), and the AT contents are 60.2/61.6% and 60.9/60.7% (Table 4).

**Table 4:** Nucleotide composition of the female and male *Solenaia carinatus* mitochondrial genomes.

	**AT%**		**GC%**		**AT Skew**		**GC Skew**	
	**F**	**M**	**F**	**M**	**F**	**M**	**F**	**M**
**Whole genome (L strand)**	60.9	61.0	39.1	39.0	0.22	0.27	-0.39	-0.38
***rrnS***	60.2	60.9	39.8	39.1	0.22	0.26	-0.14	-0.18
***rrnL***	61.6	60.7	38.4	39.3	0.19	0.27	-0.18	-0.19
**Protein-coding genes**	60.0	60.5	40.0	39.5	-0.26	-0.25	0.16	0.20
1st codon position	55.9	56.9	44.1	43.1	-0.10	-0.06	0.29	0.29
2nd codon position	59.9	60.5	40.1	39.5	-0.46	-0.41	-0.03	0.03
3rd codon position	64.0	64.1	36.0	35.9	-0.20	-0.28	0.23	0.27
**tRNA genes**	62.8	61.1	37.2	38.9	0.08	0.13	-0.03	-0.07
**Control region**	63.7	66.5	36.3	33.5	-0.05	0	-0.50	-0.43

### Unassigned regions

Both F and M genomes have many unassigned regions (27 in F and 23 in M). Among these, F *nad5*-*trnQ* and M *trnH*-*trnQ* intergenic regions (Figure 1) are the most likely candidates for the control regions (CR).

Unlike vertebrates, the CR in invertebrates is not well characterized and lacks discrete, conserved sequence blocks used in identification [64], and can greatly vary in numbers, lengths and positions. Long and highly conserved motifs with tandem repeats are likely involved in controlling replication and transcription [17] (e.g., 72,73). 

**Figure 2:**
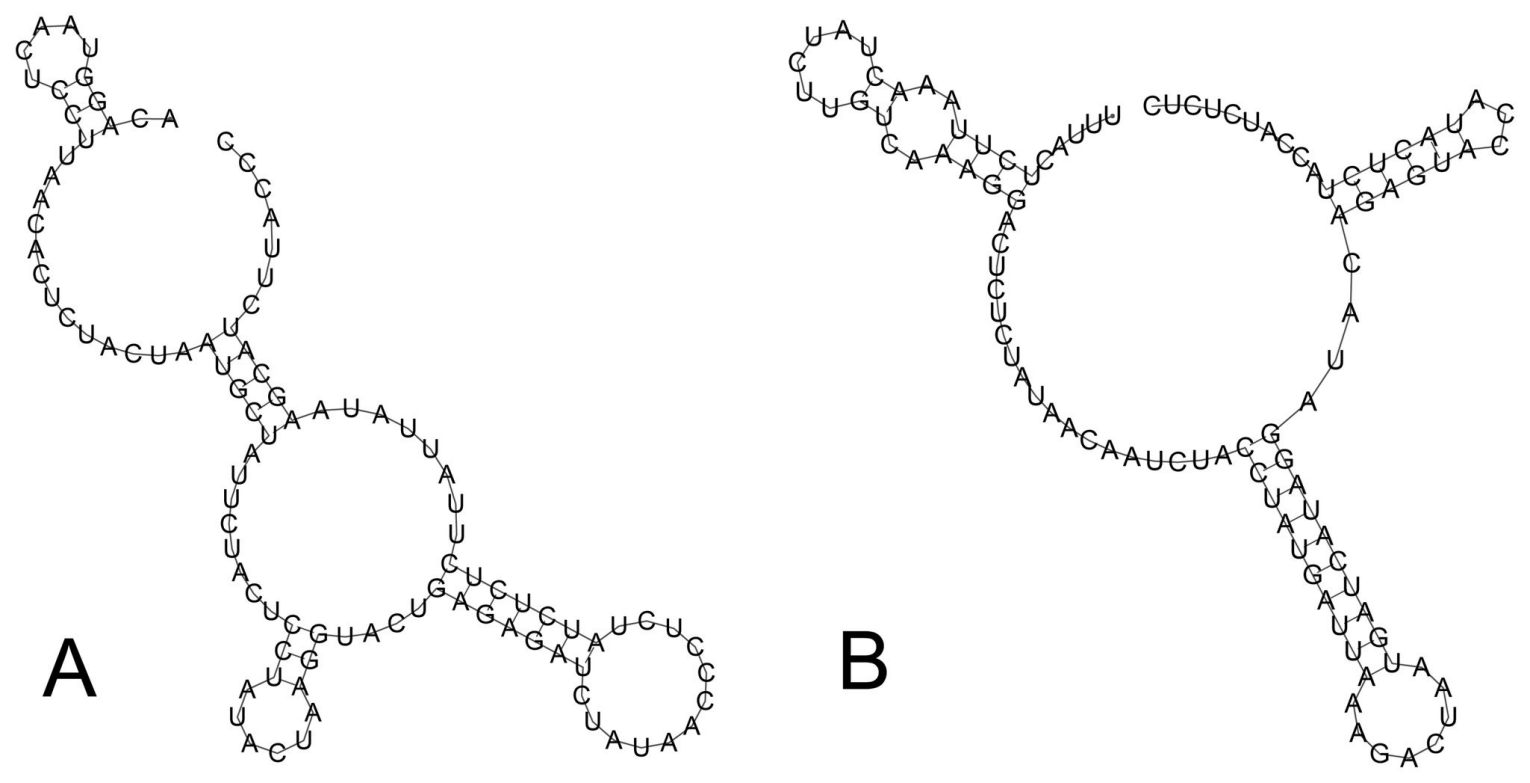
Stem-loop structures predicted for tandem repeat elements in putative control regions of female and male *Solenaia carinatus* mitochondrial genomes. (A) Female *S. carinatus* mitochondrial genome. (B) Male *S. carinatus* mitochondrial genome.

The putative control regions F *nad5*-*trnQ* and M *trnH*-*trnQ* are the largest unassigned regions (1049 and 848 bp respectively) and have relatively higher A+T content (F=63.7%, M=66.5%, Table 4) compared with other parts of the mt genome. Moreover, there are 8 tandem repeats of a 101 bp element in F *nad5*-*trnQ* region and 7 consecutive repeats of a 102 bp element in M *trnH*-*trnQ* region with both these elements have the potential to form stem-loop structures (Figure 2). Similar tandem repeat units were also found within the control regions of other unionoids [18] and other animals like nemerteans [74]. 

Hence, F *nad5*-*trnQ* and M *trnH*-*trnQ* intergenic regions of *S. carinatus* match all three criteria used to identify mitochondrial control regions: length, AT content, and the presence of tandem repeats with stem-loop structure [18].

### Base composition and codon usage

For the composition bias showing high values of C% over G%, the F and M coding strand can be considered as the light (L) strand. Though variable values of A+T content in mtDNA are common in the mollusks [53], the composition bias showing high values of A+T% over G+C%. As for *S. carinatus*, the A+T content of the L strand is also high (F = 60.9%, M = 61.0%, Table 4). The A+T content for PCGs in F and M is 60.0% and 60.5% respectively, and the third codon positions A+T composition is 64.0% and 64.1% in F and M genome respectively, consistent with the typical invertebrate bias favoring codons ending with an A or T [75]. The coding strand of PCGs displays the negative AT- and positive GC-skew, showing bias toward T over A and toward G over C, which is reflected further in the use of synonymous codons (Table S1). 

UUU (F) is the most frequent codon in both F and M mt genomes, followed by AUU (I) in F genome and GUU (V) in M genome. UUU is also the most frequent codon in other invertebrate mtDNAs [16,53,76]. Except for the stop codons, CGC is the least used codon in F and M mt genomes, which is also among the least common codon in the mtDNA of other mollusks [53,76].

### The extension of *cox2* in male mtDNA

M genomes of unionoid bivalves possess a unique, rapidly evolving coding 3' extension of *cox2* (M*cox2e*) that typically yields an ~80% increase in gene length over that in the corresponding F genomes [77,78]. 

The male *cox2* gene of *S. carinatus* shows a 543 bp extension, resulting in 79.7% 3' extension compared with the corresponding female *cox2* gene, while in other DUI animals, an extra copy of the *cox2* gene was found in the M genome of the marine mussel *Musculista senhousia* [53].

It is noteworthy that the unique extension of M*cox2* is functional. Seasonal expression of M*COX2e* in testis suggests that it plays an important role in reproduction [24,79]. M*COX2e* is localized to the inner and outer sperm mitochondrial membranes [24], and could be involved in segregation of male mitochondria to the gonad, preventing the M genome from degrading [77], which play a significant role in stability of the DUI system.

### Sequence divergences of two gender-associated mitochondrial genomes

Among species with DUI, freshwater mussels exhibit the greatest nucleotide and amino acid divergences between their maternally and paternally inherited mtDNAs and the measured divergences considerably surpass intra- or inter-species values [16]. 

In *S. carinatus*, the amino acid divergence and the nucleotide divergence between the F and M genomes are 48% and 40% respectively (Table 5). The observed conspecific genome divergence is much higher than marine mussels in nucleotide (e.g., *Musculista senhousia* (23%) [53]; *Mytilus edulis* (23%) [80]), but relatively lower than other freshwater mussels both in amino acid and nucleotide divergence respectively (e.g., *Unio japanensis* (51%, 43%), *Quadrula quadrula* (52%, 42%), *Pyganodon grandis* (51%, 43%) and *Venustaconcha ellipsiformis* (50%, 41%) [16]).

**Table 5:** Divergences in female and male *Solenaia carinatus* mitochondrial genomes.

Gene	pD ± SE^nt^	pD ± SE^aa^	Ka	Ks	Ka/Ks
*rrnS*	0.291 ± 0.017	NA	NA	NA	NA
*rrnL*	0.289 ± 0.012	NA	NA	NA	NA
*cox1*	0.299 ± 0.012	0.311 ± 0.019	0.2391	1.1643	0.2054
*cox2*	0.369 ± 0.017	0.458 ± 0.033	0.4031	1.0467	0.3851
*cox3*	0.351 ± 0.019	0.397 ± 0.030	0.3347	1.2319	0.2717
*nad1*	0.433 ± 0.016	0.470 ± 0.028	0.4418	2.3138	0.1909
*nad2*	0.496 ± 0.015	0.631 ± 0.026	0.6806	1.4316	0.4754
*nad3*	0.407 ± 0.025	0.513 ± 0.046	0.5059	0.9738	0.5195
*nad4*	0.408 ± 0.012	0.500 ± 0.022	0.4699	1.1809	0.3979
*nad4L*	0.466 ± 0.029	0.660 ± 0.047	0.6874	0.9322	0.7374
*nad5*	0.432 ± 0.011	0.556 ± 0.020	0.5335	1.1510	0.4635
*nad6*	0.469 ± 0.023	0.596 ± 0.039	0.6197	1.1898	0.5209
*cob*	0.385 ± 0.013	0.411 ± 0.024	0.3465	2.3282	0.1488
*atp6*	0.438 ± 0.021	0.498 ± 0.032	0.4962	1.5631	0.3175
*atp8*	0.508 ± 0.037	0.638 ± 0.062	0.7725	1.0234	0.7548
All proteins*	0.404 ± 0.004	0.481 ± 0.008	0.4402	1.3230	0.3327

pD = p-Distances.

SE = Standard Error.pD ± SE^nt^ and pD ± SE^aa^ are pD ± SE at nucleotide and amino acid level respectively.Ka and Ks = divergence of protein genes in non-synonymous (Ka) and synonymous (Ks) sites respectively.Ka/Ks = ratio values between Ka and Ks.NA = Not Available*: Female-specific FORF and male-specific MORF were excluded from the computation.

Since existing universal primers were designed based on maternally inherited mitochondrial genomes, there are a limited number of universal primers (e.g., universal primers for cox1 and 16S rRNA) could apply to the M genome besides F genome in the freshwater mussels. Hence, knowing the sequence divergences among genes in these two sex-linked mitogenomes will help us select and develop suitable genetic markers, especially the M-specific marker, for population structure analysis and gene flow measurement.

Comparing these two mtDNAs, the most conserved protein-coding genes are *cox1*, *cox3* and *cob*, while the least conserved are *nad4L*, *atp8* and *nad2* (amino acid divergence; Table 5). Synonymous (Ks) and non-synonymous (Ka) values between the two gender-associated mitogenomes vary among genes (Table 5). *cox1* has lowest Ka (0.2391) and modest Ks (1.1643), suggesting this gene may be under higher selective pressure. In *cob* gene, Ka is lower than average but the Ks is the highest with the Ka/Ks value the lowest (0.1488). For *nad1* gene, the Ks (2.3138) is the second highest, whereas Ka is modest (0.4418).

### Phylogenetic relationships of Unionoida inferred from the mitochondrial genome sequences

The latest classification of Unionidae left out the hinge teeth as a higher-level taxonomic characteristic, and deemed that the subfamily Anodontinae should be demoted to a tribe within Unioninae owing to the shared hooked type and subtriangular external shape of the glochidia [81-83]. However, morphological characteristics of glochidia in these two subfamilies are not in complete consensus. According to Wu [13], the Anodontinae glochidia are elongated triangular (except for the non-parasitic *Anodonta arcaeformis* that is semicircular) in external shape and have pores on the shell surface, while the Unioninae glochidia are mostly wide triangular with pits on the shell. Therefore, we are more inclined to treat the tribe Anodontini as a subfamily paralleled to Unioninae, in accordance with traditional classifications.

**Figure 3:**
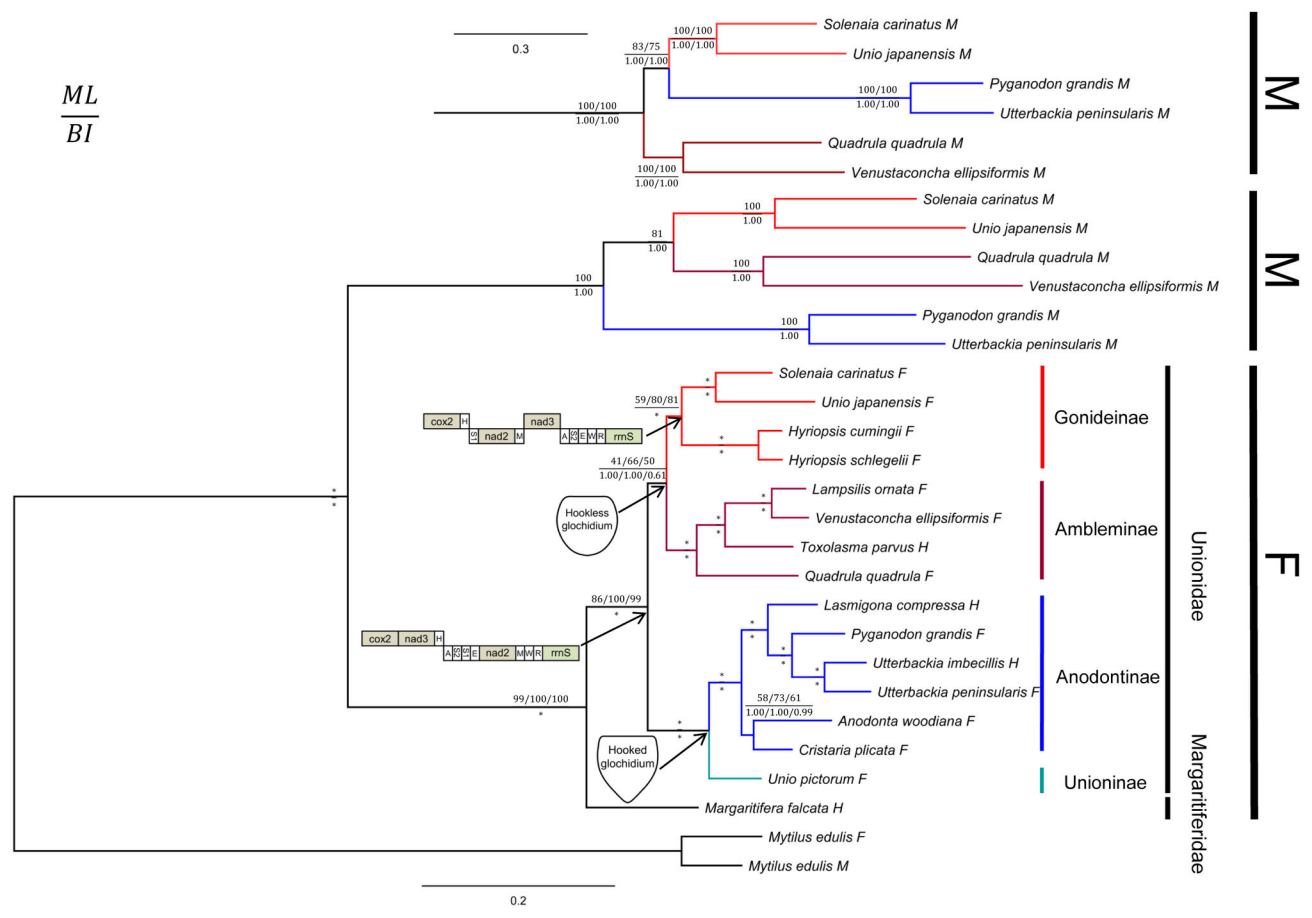
Phylogenetic trees of freshwater mussels inferred from 12 mitochondrial protein-coding gene sequences (except *atp8* and gender-specific ORFs). AA dataset have the best topology. Alternative M clade topology of PCG123 and PCG12 was correspondingly shown on the topside of the tree. Numbers from left to right are from AA, PCG123 and PCG12 alignments respectively. Numbers close to the branching points are ML bootstrap support values (above) and Bayesian posterior probabilities (below). An asterisk indicates that all three dataset-based nodal support values are 100%. The glochidium hook type and the comparison of F genome gene order were also annotated in the corresponding clades.

We reconstructed the phylogenetic relationships of freshwater mussels using both F and M genomes of *S. carinatus* combined with all 20 currently available complete F and M mitochondrial genomes of 15 unionoid bivalves. Three datasets: 1) amino acid sequences of 12 protein-coding genes (AA); 2) nucleotide sequences of 12 protein-coding genes (PCG123); and 3) nucleotide sequences of the first and second codon positions of protein-coding genes (PCG12) were used. ML and BI trees based on all these three datasets have largely congruent topology and were statistically well supported by high bootstrap and posterior probability values in most nodes (Figure 3, Figure S1). Three datasets all have strong support (ML = 100, BI = 1.00) for the reciprocal monophyly of F and M clades, in accordance with the previous viewpoint [16]. Branch lengths indicate that unionoid M genomes have significantly higher substitution rate compared with the corresponding F genomes. The faster evolving nature of M genome may be due to higher rate of M mtDNA replication during spermatogenesis, smaller effective population size for the M genomes, positive selection for the M genomes, relaxed selective constrains for the M genomes, or a combination of these processes [84]. Adding M genome sequences to the traditional routine of F genome sequence phylogenetic analyses could often result in more robust evolutionary relationships than those provided by analyses of F sequences alone [42].

With currently available mitogenomes, all three datasets support the monophyly of four Unionidae subfamily (i.e., Unioninae, Anodontinae, Ambleminae, and Gonideinae) in both F and M (lack the only Unioninae species) clades by both ML and BI methods. 

The AA dataset does not have strong ML support (ML = 59) but have robust BI support (BI = 1.00) for the monophyly of Gonideinae in F clade. The nucleotide sequence datasets (PCG123 and PCG12 datasets), however, do have high ML support and strong BI support (ML = 80, 81; BI = 1.00, 1.00). Additionally, the M genome sequences support the monophyly of Gonideinae with 100% by BI and ML analyses regardless of the dataset used.

The phylogenetic analyses of F genome sequences reveal: (Unioninae + Anodontinae) + (Ambleminae + Gonideinae) of Unionidae. Though the sister group relationship of Ambleminae and Gonideinae is not highly supported by ML in the F clade, it is consistent with the morphological taxonomy based on glochidial hook type [13,14,85,86] (Figure 3).

Amino acid sequences (AA dataset) of the M genome show the sister relationship between Ambleminae and Gonideinae congruent with F genome. However, nucleotide sequences (PCG123 and PCG12 datasets) place Ambleminae being sister to (Unioninae + Gonideinae) in the M clade, resulting from long-branch attraction artifacts, which could be solved by more representative and denser taxon availability of M-type mt genome sequences in the future. 

Excluding third codon positions from the analysis (PCG12) significantly decreases support for the sister group relationship of Ambleminae and Gonideinae (ML = 66-50, BI = 1.00-0.61) in the F clade, and cannot solve the long-branch attraction due to the sparse M-type mt genome sampling in the present study.

*S. carinatus* belongs to the subfamily Gonideinae as revealed by all tree topologies obtained from both F and M genomes (ML = 100%, BI = 1.00), and shares closer relationships with the Asian unionid *Unio japanensis*, *Hyriopsis cumingii* and *H. schlegelii* (Figure 3). Analyses of partial 16S rRNA, however, indicated that *Solenaia* together with *Lamprotula*, *Hyriopsis*, and *Ptychorhynchus* were the Asian 'amblemines' [37]. It seems that the short sequence of partial 16S rRNA did not contain enough phylogenetic information to resolve the relationships correctly so that the Gonideinae was wrongly nested within Ambleminae. In the Gonideinae, another intriguing mussel is the western North American *Gonidea angulata*. *Gonidea* is a monotypic genus, and morphologically and anatomically more similar to freshwater mussels of Southeast Asia than that of North America [81]. We hypothesize that there is an affinity of *Gonidea* with East Asian *Solenaia*, especially the species *S. triangularis*, for the strikingly morphological and habitual similarities, which was also proposed by Simpson [87]. Further molecular phylogeny studies with denser representative taxa and more mitochondrial genomes are needed to test this hypothesis.

### Gene order and its phylogenetic implications

Gene order of the animal mtDNA is particularly interesting for problems of deep-level phylogenetic relationships [88] as it is characterized by a large number of states and often remains unchanged over long evolutionary periods [15,89]. Bivalves show frequent and extensive mtDNA variability at intra-genus level, and the mtDNA plasticity is mainly due to variation in number and location of tRNA genes, which rarely concerns protein-coding and rRNA genes [15].

*S. carinatus* F and M genome gene arrangements are notably different from each other. The transposition of *trnH* and the gene order inversion of *trnD*-*atp8* contribute to the differences of two gender-associated genomes (Figure 1), consistent with other freshwater mussels [18].

In addition, the rearrangement between *cox2* and *rrnS* is also responsible for the remarkable structural differences between *S. carinatus* F and M genomes (Figure 1 and Figure 3). Most unionoid taxa (including both F and M mt genomes) have a congruent order and strand orientation of 13 typical PCGs and 2 rRNAs: *cox1-cox2-nad3-nad2-rrnS-rrnL-cob-nad5-nad1-nad6-nad4-nad4L-atp8-atp6-cox3*. However, the PCG order of F genome in *S. carinatus* differs substantially from corresponding M genome with the inversion of the relative position of *nad2* and *nad3*. 

This F genome *cox2*-*rrnS* gene arrangement is not unique for *S. carinatus*, but is also shown to exist in other available Gonideinae species (i.e., *Unio japanensis*, *Hyriopsis cumingii*, *H. schlegelii* (Table 2), and *Lamprotula leai* [90]), which suggests that this unique gene order is a synapomorphy for female lineage of the subfamily Gonideinae, and the unique apomorphic PCG arrangement supports its well-established monophyly (Figure 3).

## Conclusion

The newly sequenced maternal and paternal mitochondrial genomes of the endangered freshwater mussel *Solenaia carinatus* diverge by about 48% in amino acid sequence and 40% in nucleotide sequence. Gene arrangements between these two gender-associated genomes are notably different, and the F genome *cox2-rrnS* gene arrangement is considered a genome-level synapomorphy for female lineage of Gonideinae. Combined with morphological characteristics of glochidia, phylogenetic analyses in the context of complete female and male mitochondrial genomes from 22 freshwater mussel mt genomes strongly indicate *S. carinatus* belongs to the subfamily Gonideinae and support the classification of the sampled Unionidae species into four subfamilies: Gonideinae, Ambleminae, Anodontinae, and Unioninae.

For phylogenetic analyses of freshwater mussel mt genome data, simply excluding the third codon positions of the protein-coding genes is detrimental to phylogenetic reconstruction, and the conservative amino acid sequences provide the best topologies in both F and M clades, given the underrepresentation in M genome of unionoids.

Glochidia, which transport both maternal and paternal mitochondrial genomes, have limited dispersal abilities mainly restricted to fish patterns; the M-type mitochondria, however, can move as sperm downstream, having greater gene mobility [91]. Furthermore, the M genome evolves much faster than the F genome resulting in higher polymorphism. Consequently, it is expected to be easier to deduce population structure using M instead of F mtDNA data. On the other hand, selective sweeps happen periodically and repeatedly in M lineages while less frequently in F lineages [19]. Therefore, the DUI system can be used as an ideal model to study the mitogenome evolution.

## Supporting Information

Figure S1 **Six phylogenetic trees of freshwater mussels inferred from 12 mitochondrial protein-coding gene sequences (except *atp8* and gender-specific ORFs).**
(PDF)

Table S1 **Codon usage in female and male *Solenaia carinatus* mitochondrial genomes.**
(DOCX)
