# Supplementary figures and images for: The Complete Maternally and Paternally Inherited Mitochondrial Genomes of the Endangered Freshwater Mussel Solenaia carinatus (Bivalvia: Unionidae) and Implications for Unionidae Taxonomy
Source: PLoS One. 2013 Dec 17;8(12):e84352. doi: 10.1371/journal.pone.0084352 (PMC3866145; doi:10.1371/journal.pone.0084352)

BI  
AA

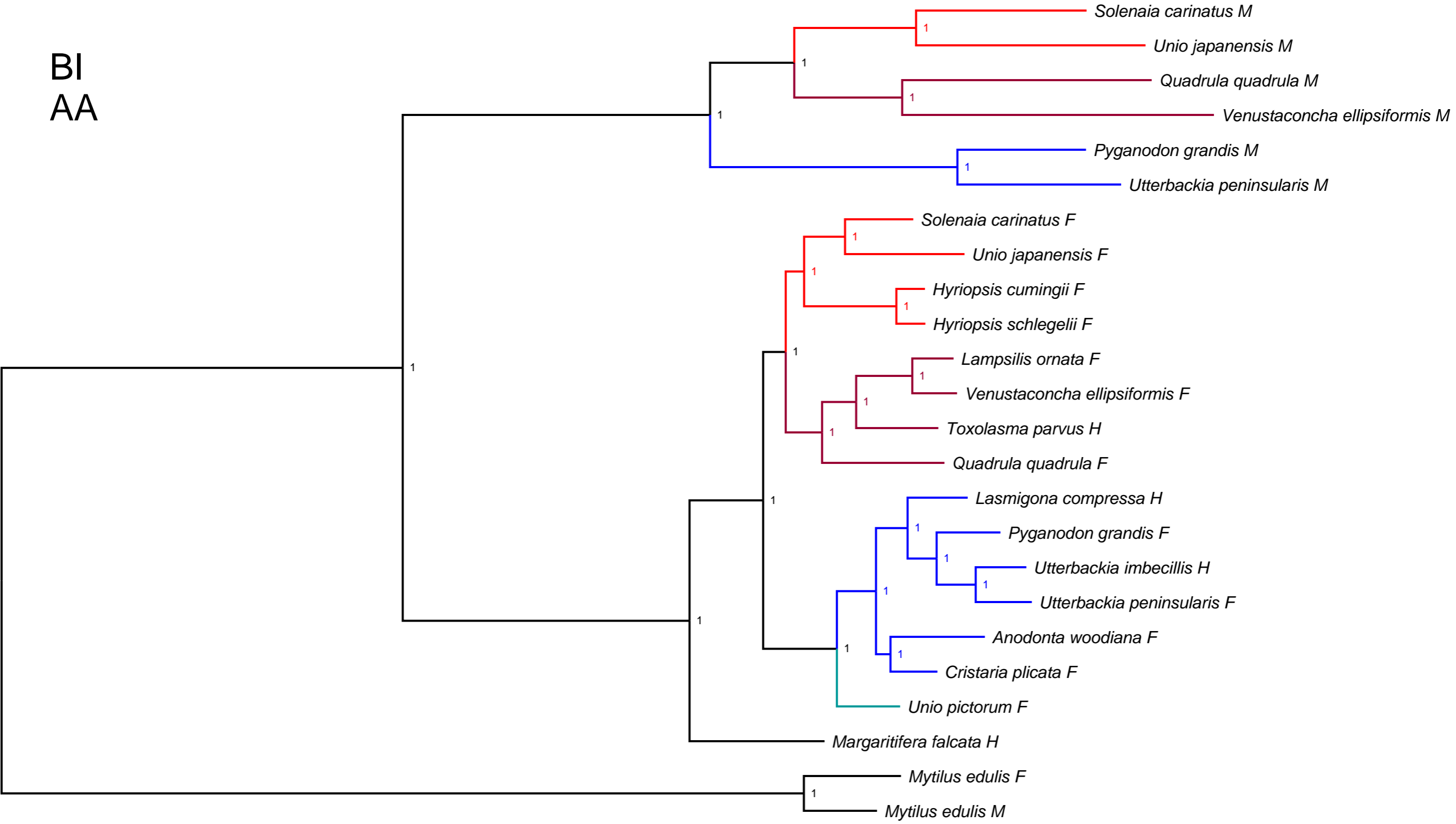

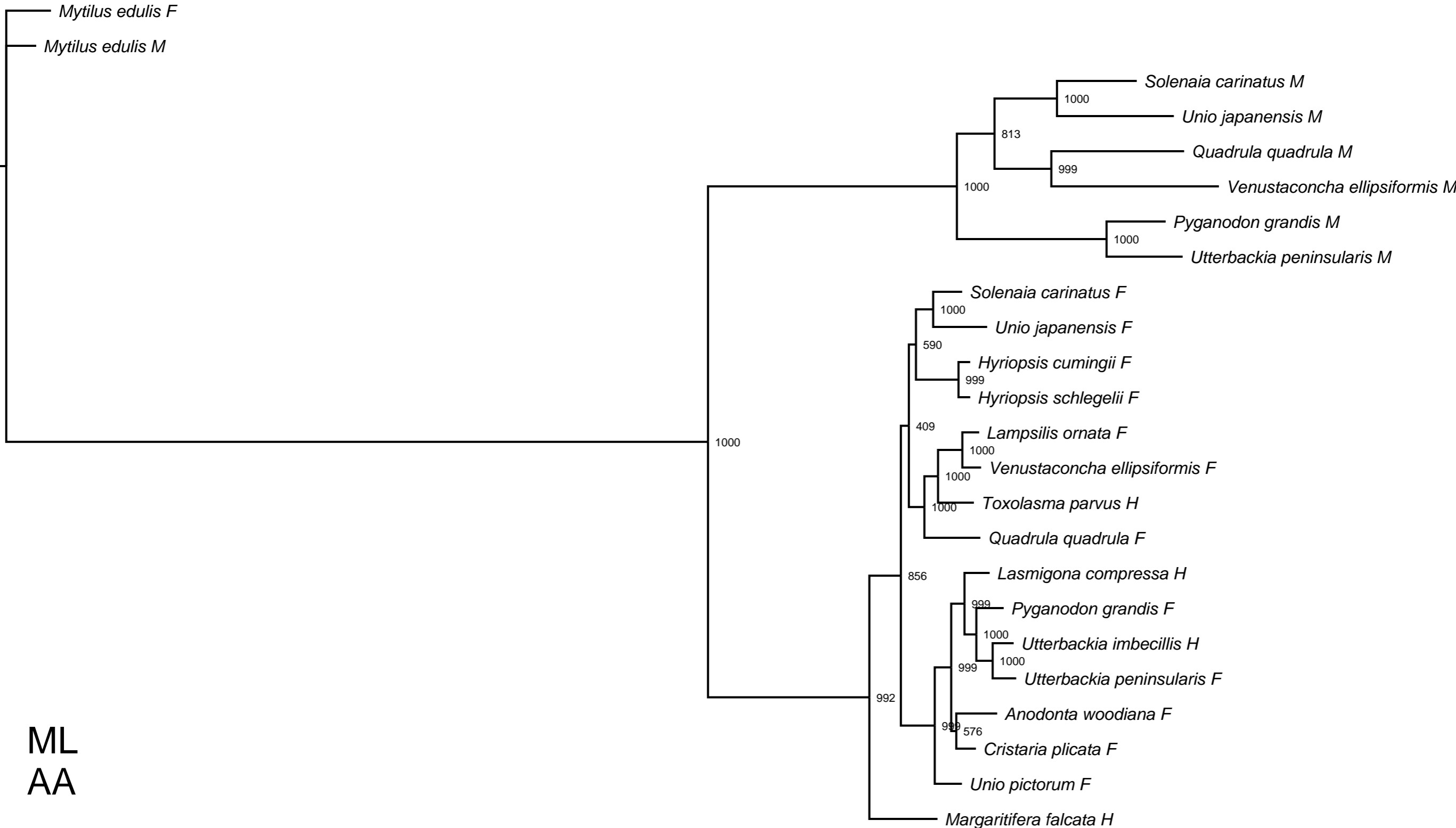

BI  
PCG123

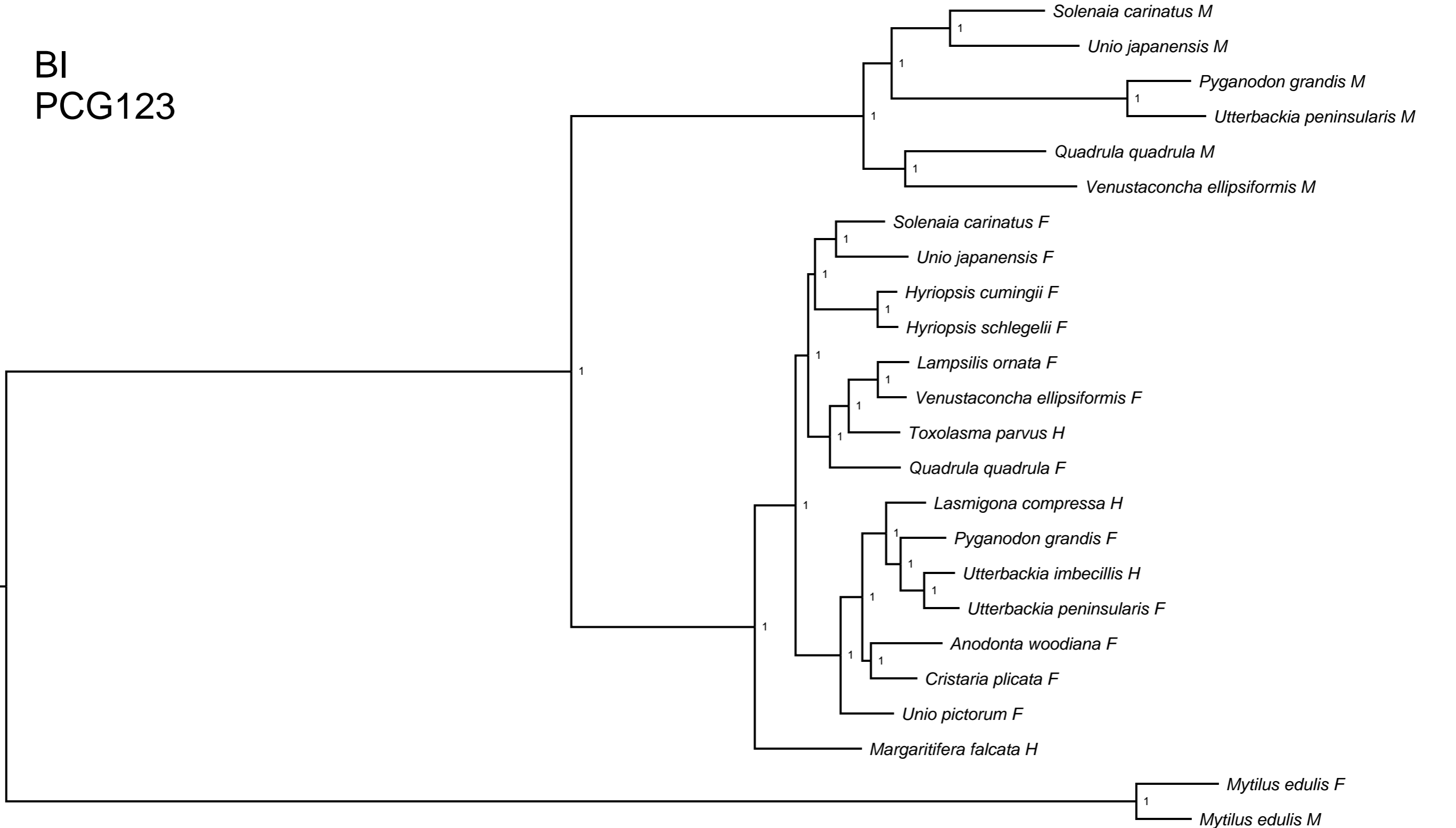

0.5

ML  
PCG123

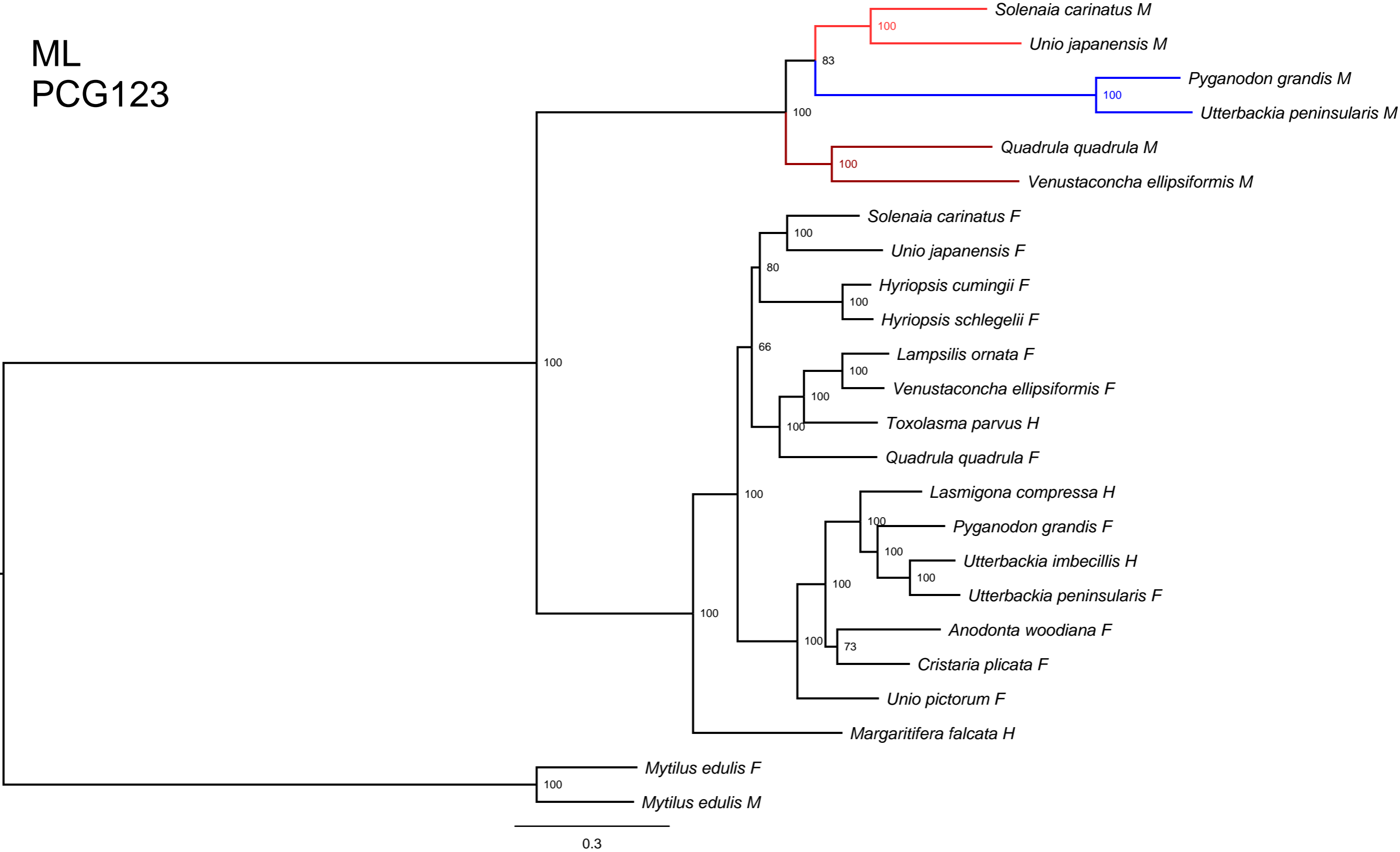

BI  
PCG12

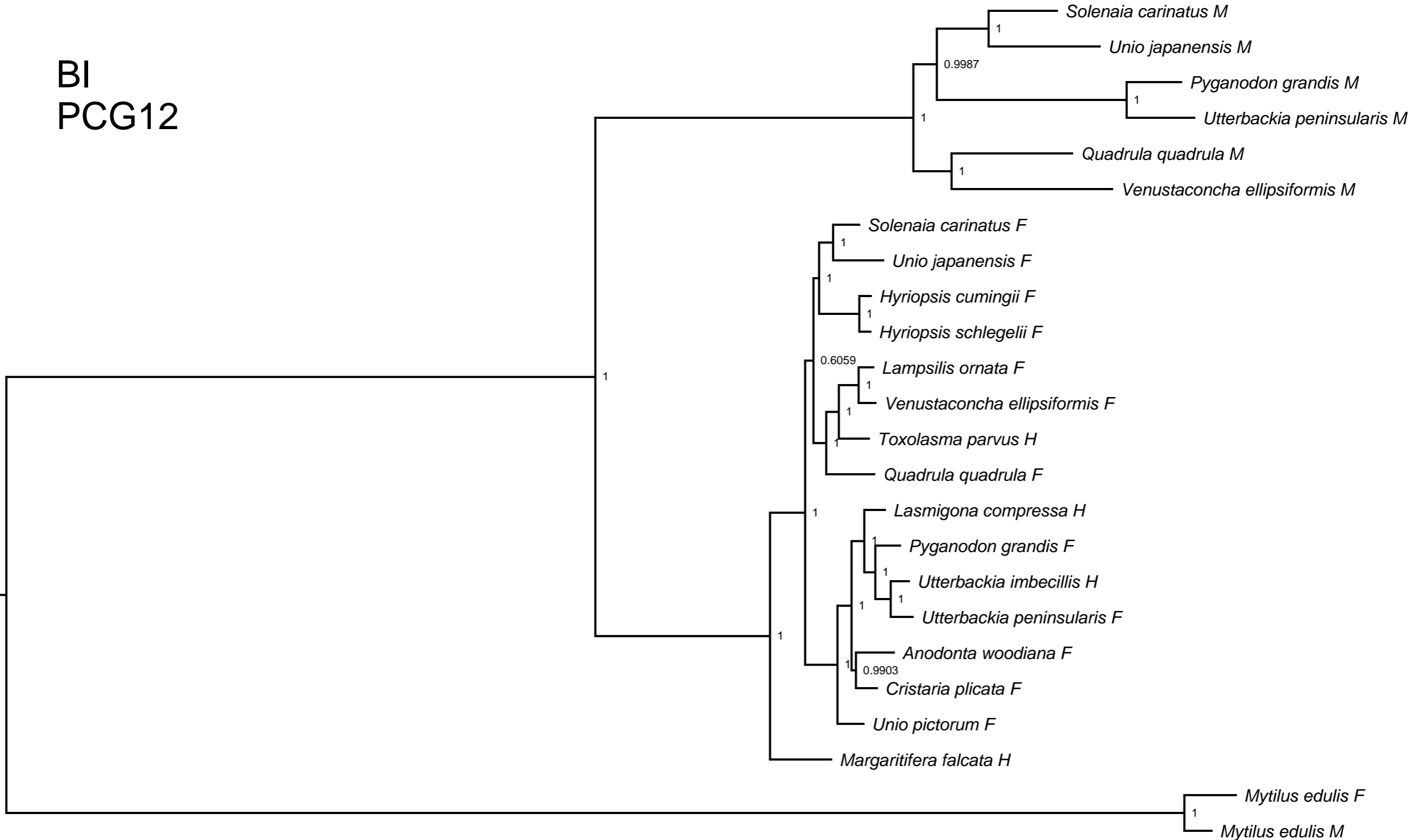

ML  
PCG12

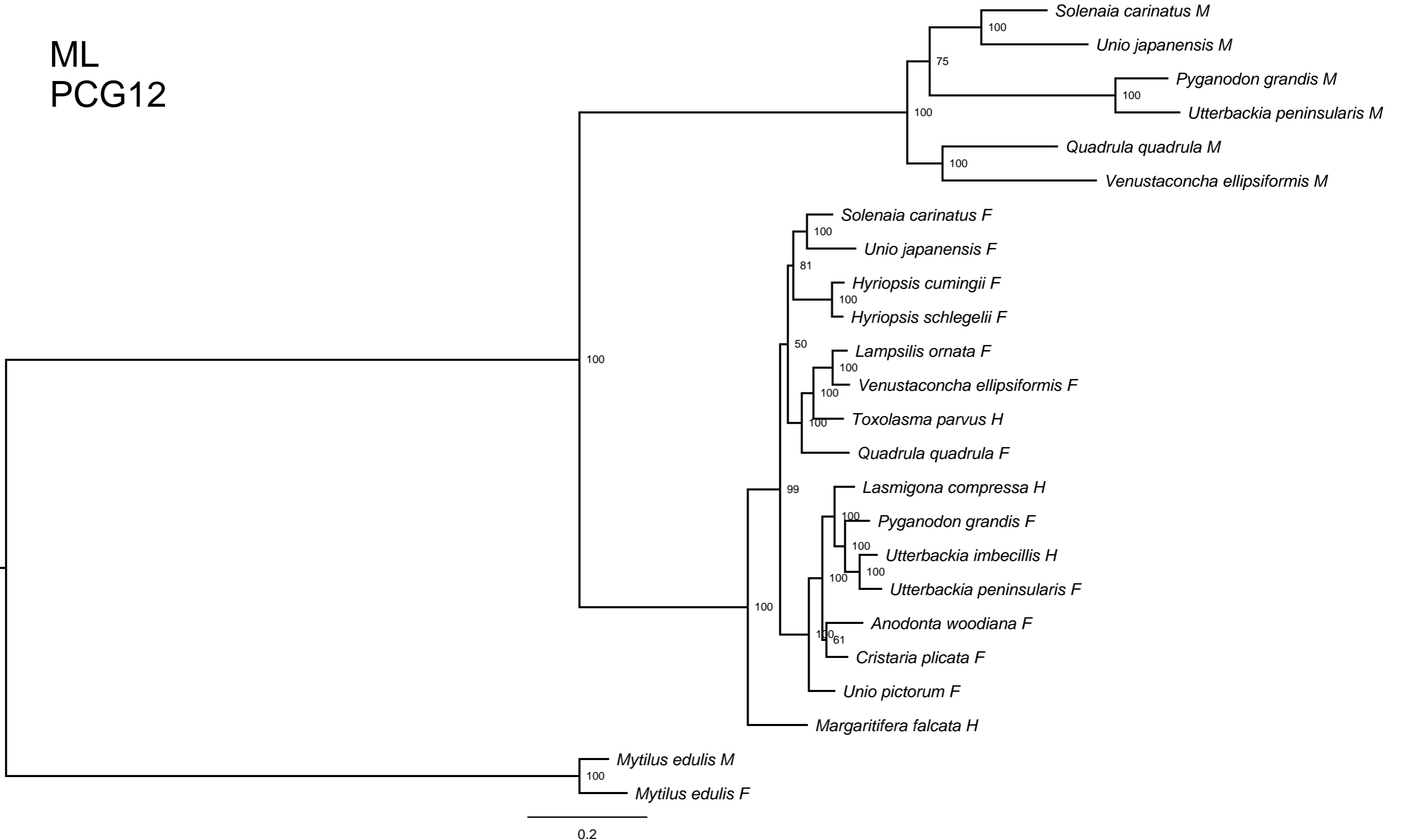

Supplement: Figure S1 — Six phylogenetic trees of freshwater mussels inferred from 12 mitochondrial protein-coding gene sequences (except atp8 and gender-specific ORFs). (PDF) [file pone.0084352.s001.pdf]
