# Supplementary material for: The Complete Maternally and Paternally Inherited Mitochondrial Genomes of the Endangered Freshwater Mussel Solenaia carinatus (Bivalvia: Unionidae) and Implications for Unionidae Taxonomy
Source: PLoS One. 2013 Dec 17;8(12):e84352. doi: 10.1371/journal.pone.0084352 (PMC3866145; doi:10.1371/journal.pone.0084352)
Supplement: Table S1 — Codon usage in female and male Solenaia carinatus mitochondrial genomes. (DOCX) [file pone.0084352.s002.docx]

**Table S1**. Codon usage in Female and Male *Solenaia carinatus* mitochondrial genomes.

| FEMALE | | | | | | | | | | | |
| --- | --- | --- | --- | --- | --- | --- | --- | --- | --- | --- | --- |
| **Codon** | **Count** | **RSCU** | **Codon** | **Count** | **RSCU** | **Codon** | **Count** | **RSCU** | **Codon** | **Count** | **RSCU** |
| UUU(F) | 241 | 1.62 | UCU(S) | 115 | 2.37 | UAU(Y) | 88 | 1.36 | UGU(C) | 38 | 1.43 |
| UUC(F) | 56 | 0.38 | UCC(S) | 36 | 0.74 | UAC(Y) | 41 | 0.64 | UGC(C) | 15 | 0.57 |
| UUA(L) | 165 | 1.71 | UCA(S) | 45 | 0.93 | UAA(*) | 7 | 1 | UGA(W) | 62 | 1.08 |
| UUG(L) | 182 | 1.89 | UCG(S) | 23 | 0.47 | UAG(*) | 7 | 1 | UGG(W) | 53 | 0.92 |
| CUU(L) | 75 | 0.78 | CCU(P) | 50 | 1.3 | CAU(H) | 58 | 1.47 | CGU(R) | 14 | 0.9 |
| CUC(L) | 37 | 0.38 | CCC(P) | 24 | 0.62 | CAC(H) | 21 | 0.53 | CGC(R) | 6 | 0.39 |
| CUA(L) | 89 | 0.92 | CCA(P) | 65 | 1.69 | CAA(Q) | 43 | 1.23 | CGA(R) | 31 | 2 |
| CUG(L) | 30 | 0.31 | CCG(P) | 15 | 0.39 | CAG(Q) | 27 | 0.77 | CGG(R) | 11 | 0.71 |
| AUU(I) | 183 | 1.46 | ACU(T) | 70 | 1.47 | AAU(N) | 53 | 1.13 | AGU(S) | 38 | 0.78 |
| AUC(I) | 67 | 0.54 | ACC(T) | 42 | 0.88 | AAC(N) | 41 | 0.87 | AGC(S) | 24 | 0.49 |
| AUA(M) | 90 | 1.07 | ACA(T) | 56 | 1.18 | AAA(K) | 55 | 1.33 | AGA(S) | 51 | 1.05 |
| AUG(M) | 78 | 0.93 | ACG(T) | 22 | 0.46 | AAG(K) | 28 | 0.67 | AGG(S) | 57 | 1.17 |
| GUU(V) | 161 | 1.75 | GCU(A) | 115 | 2.04 | GAU(D) | 47 | 1.4 | GGU(G) | 118 | 1.4 |
| GUC(V) | 28 | 0.3 | GCC(A) | 44 | 0.78 | GAC(D) | 20 | 0.6 | GGC(G) | 28 | 0.33 |
| GUA(V) | 85 | 0.92 | GCA(A) | 40 | 0.71 | GAA(E) | 31 | 0.73 | GGA(G) | 58 | 0.69 |
| GUG(V) | 94 | 1.02 | GCG(A) | 26 | 0.46 | GAG(E) | 54 | 1.27 | GGG(G) | 133 | 1.58 |
| MALE | | | | | | | | | | | |
| **Codon** | **Count** | **RSCU** | **Codon** | **Count** | **RSCU** | **Codon** | **Count** | **RSCU** | **Codon** | **Count** | **RSCU** |
| UUU(F) | 275 | 1.65 | UCU(S) | 119 | 2.18 | UAU(Y) | 94 | 1.27 | UGU(C) | 77 | 1.66 |
| UUC(F) | 59 | 0.35 | UCC(S) | 38 | 0.7 | UAC(Y) | 54 | 0.73 | UGC(C) | 16 | 0.34 |
| UUA(L) | 154 | 1.58 | UCA(S) | 37 | 0.68 | UAA(*) | 4 | 0.57 | UGA(W) | 50 | 0.85 |
| UUG(L) | 176 | 1.8 | UCG(S) | 19 | 0.35 | UAG(*) | 10 | 1.43 | UGG(W) | 67 | 1.15 |
| CUU(L) | 87 | 0.89 | CCU(P) | 62 | 1.54 | CAU(H) | 52 | 1.33 | CGU(R) | 27 | 1.52 |
| CUC(L) | 38 | 0.39 | CCC(P) | 24 | 0.6 | CAC(H) | 26 | 0.67 | CGC(R) | 7 | 0.39 |
| CUA(L) | 94 | 0.96 | CCA(P) | 54 | 1.34 | CAA(Q) | 38 | 1.1 | CGA(R) | 23 | 1.3 |
| CUG(L) | 37 | 0.38 | CCG(P) | 21 | 0.52 | CAG(Q) | 31 | 0.9 | CGG(R) | 14 | 0.79 |
| AUU(I) | 184 | 1.52 | ACU(T) | 66 | 1.39 | AAU(N) | 58 | 1.04 | AGU(S) | 81 | 1.48 |
| AUC(I) | 58 | 0.48 | ACC(T) | 52 | 1.09 | AAC(N) | 54 | 0.96 | AGC(S) | 24 | 0.44 |
| AUA(M) | 117 | 1.11 | ACA(T) | 60 | 1.26 | AAA(K) | 67 | 1.02 | AGA(S) | 43 | 0.79 |
| AUG(M) | 94 | 0.89 | ACG(T) | 12 | 0.25 | AAG(K) | 64 | 0.98 | AGG(S) | 76 | 1.39 |
| GUU(V) | 214 | 2.16 | GCU(A) | 115 | 2.02 | GAU(D) | 65 | 1.6 | GGU(G) | 123 | 1.45 |
| GUC(V) | 14 | 0.14 | GCC(A) | 46 | 0.81 | GAC(D) | 16 | 0.4 | GGC(G) | 16 | 0.19 |
| GUA(V) | 84 | 0.85 | GCA(A) | 47 | 0.82 | GAA(E) | 35 | 0.67 | GGA(G) | 51 | 0.6 |
| GUG(V) | 84 | 0.85 | GCG(A) | 20 | 0.35 | GAG(E) | 69 | 1.33 | GGG(G) | 150 | 1.76 |

*: stop codon.

RSCU: Relative synonymous codon usage is the number of times a particular codon is defined relative to the number of times codon would be observed in the absence of any codon usage bias [[1](#_ENREF_1)].

References

1. Sharp PM, Cowe E, Higgins DG, Shields DC, Wolfe KH, et al. (1988) Codon usage patterns in *Escherichia coli, Bacillus subtilis, Saccharomyces cerevisiae, Schizosaccharomyces pombe, Drosophila melanogaster* and *Homo sapiens*: a review of the considerable within-species diversity. Nucleic Acids Research 16: 8207-8211.
